# Pan-Cancer Analysis Identifies a Ras-Related GTPase as a Potential Modulator of Cancer

**DOI:** 10.3390/ijms26094419

**Published:** 2025-05-06

**Authors:** Hsiang-Yin Hsueh, Kristyn Gumpper-Fedus, Jelmer W. Poelstra, Kenneth L. Pitter, Zobeida Cruz-Monserrate

**Affiliations:** 1 Department of Internal Medicine, Division of Gastroenterology, Hepatology, and Nutrition, The Ohio State University Wexner Medical Center, Columbus, OH 43210, USA; hsiangyin.hsueh@osumc.edu (H.-Y.H.); kristyn.gumpper-fedus@osumc.edu (K.G.-F.); 2 The James Comprehensive Cancer Center, The Ohio State University, Columbus, OH 43210, USA; kenneth.pitter@osumc.edu; 3 The Ohio State University Molecular, Cellular, and Developmental Biology Program, The Ohio State University, Columbus, OH 43210, USA; 4 Molecular and Cellular Imaging Center (MCIC), College of Food, Agricultural, and Environmental Sciences, The Ohio State University, Wooster, OH 44691, USA; poelstra.1@osu.edu; 5 Department of Radiation Oncology, The Ohio State University Wexner Medical Center, James Comprehensive Cancer Center, Columbus, OH 43210, USA

**Keywords:** RASD1, TCGA, cancer prognosis, immune infiltration

## Abstract

Ras signaling regulates many cellular processes in cancer development. While well-known Ras-related oncogenes, such as KRAS, have been extensively explored, the role of other Ras-related genes in cancer remains poorly studied. Dexamethasone-induced Ras-related protein 1 (RASD1), a member of the Ras superfamily, is widely expressed across various tissues and is involved in inhibiting cell growth and inducing apoptosis, suggesting a potential role as a tumor suppressor. Here, we investigated RASD1 expression across multiple tissues and cancers, utilizing data from The Cancer Genome Atlas (TCGA), Human Protein Atlas, and Genotype-Tissue Expression (GTEx) databases. Our analysis revealed a significant downregulation of RASD1 mRNA expression in several cancer types compared to normal tissues, correlating with low levels of promoter methylation. Interestingly, high RASD1 expression correlated with a favorable prognosis in multiple cancers. Immune cell infiltration analysis indicated that elevated RASD1 expression is associated with an increased infiltration of CD4^+^ T cells and myeloid-derived dendritic cells in cancer. Furthermore, the expression of genes exhibiting similar expression patterns as RASD1 suggest that RASD1 may play a role in interleukin-4-mediated apoptosis and could regulate the transcription of the phosphatase and tensin homolog (PTEN) gene. Overall, these findings suggest that RASD1 may modulate immune signaling and tumor suppressive pathways.

## 1. Introduction

Ras signaling involves a complex network of cellular events in cancer biology, including differentiation, proliferation, migration, invasion, and survival [1]. Mutations in Ras genes can result in the constitutive activation of downstream signaling cascades, like the mitogen-activated protein kinase (MAPK) and the phosphoinositide 3-kinase (PI3K)/protein kinase B (AKT) pathways. These activated pathways promote tumorigenesis, cancer initiation, and resistance to apoptosis and chemotherapy [2]. While Ras oncogenes like the Kirsten rat sarcoma viral oncogene homolog (KRAS) are well studied in cancer, our understanding of other Ras family proteins remains incomplete [3]. Investigating these lesser-known Ras proteins presents an opportunity to deepen our knowledge of Ras signaling and its broader impacts on cancer [4].

Dexamethasone-induced Ras-related protein 1 (RASD1, DEXRAS1, or AGS1) is a small GTPase within the Ras superfamily [5]. RASD1 is involved in various physiological processes: in neurons, it acts as a nitric oxide effector and inhibits the adaptor protein FE65-APP-mediated signaling [6,7]; in mice, RASD1 knockout prevents diet-induced obesity [8]. However, the role of RASD1 expression in cancer remains relatively unexplored, with limited in vitro studies suggesting its potential anti-tumor effects. For instance, RASD1 suppresses cell growth in a lung cancer cell line [9] and inhibits glioma invasion and migration by inactivating the AKT/mammalian target of rapamycin (mTOR) pathways [10]. Additionally, the induction of RASD1 expression by formononetin and its metabolite calycosin promotes apoptosis in breast and prostate cancer cell lines [11,12]. Moreover, hsa-miR-375/RASD1 signaling may serve as a predictive marker for local relapse in early breast cancer [13]. Despite these findings, there remains a need for a comprehensive exploration of RASD1′s role across various cancer types to fully understand its potential role in cancer.

Pan-cancer analysis is an integrative approach used to identify common and unique molecular alterations, biological pathways, and therapeutic targets across different cancer types. This methodology has become transformative in cancer research, offering a comprehensive view of the molecular landscape across cancers using multiple omics data [14,15]. Here, we systematically analyzed RASD1 expression patterns in both tumor and normal tissues, utilizing publicly available datasets such as The Cancer Genome Atlas (TCGA) [14], Genotype-Tissue Expression (GTEx) [16], and The Human Protein Atlas [17]. We investigated potential associations between RASD1 mRNA and protein expression levels, gene mutations, methylation patterns, overall cancer survival, immune cell infiltration in tumors, and pathway regulation across multiple cancers.

## 2. Results

### 2.1. RASD1 Is Expressed in Normal and Cancer Tissues but Is Generally Downregulated in Cancer

**Figure 1:**
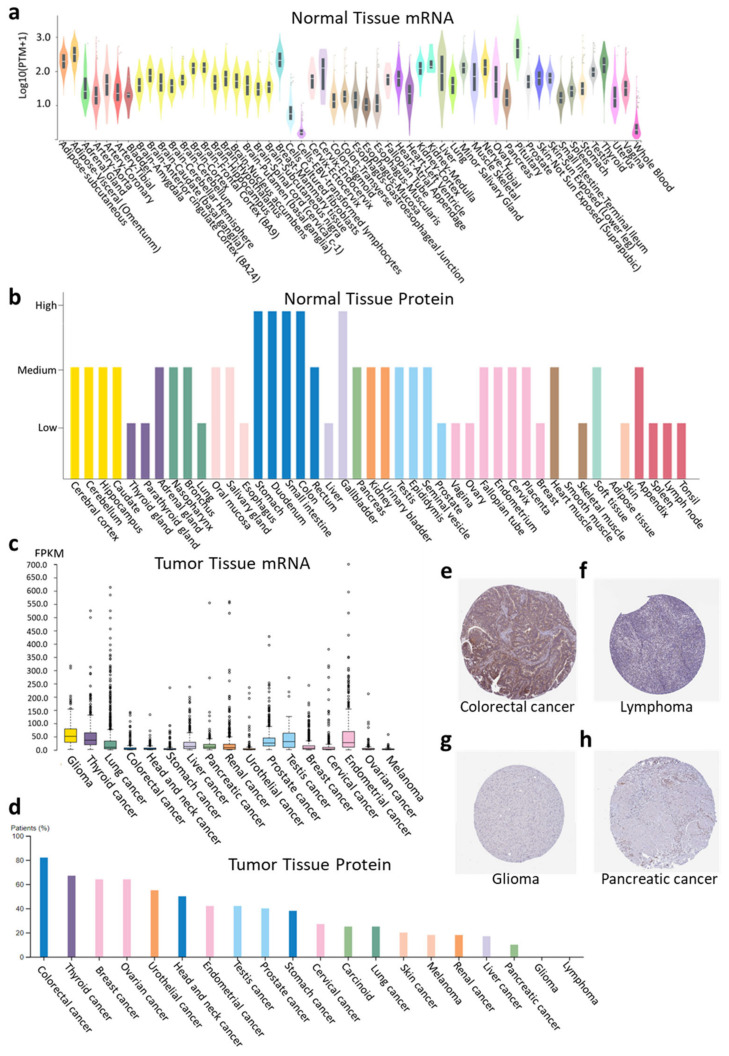
RASD1 expression pattern in normal and cancer tissues. (**a**) RASD1 mRNA expression in normal tissues from GTEX data. (**b**) Protein expression in organs from Human Protein Atlas. (**c**) RNA and (**d**) Protein expression summary of RASD1 in cancers from the Human Protein Atlas. Representative histological images of RASD1 in (**e**) colorectal cancer, (**f**) lymphoma, (**g**) glioma, and (**h**) pancreatic cancer.

**Figure 2:**
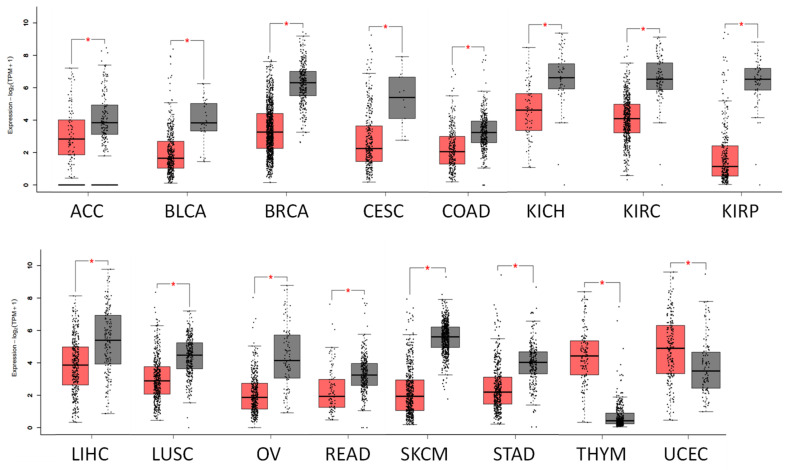
RASD1 expression is lower in most cancer types compared to normal tissue. Selected box plots of differential RASD1 mRNA expression from GEPIA2. Red: tumor, gray: normal. The normal group included normal tissue in TCGA and GTEX databases * *p* <0.05ACC: Adrenocortical carcinoma, BLCA: Bladder Urothelial Carcinoma, BRCA: Breast invasive carcinoma, CESC: Cervical squamous cell carcinoma and endocervical adenocarcinoma, COAD: Colon adenocarcinoma, KICH: Kidney Chromophobe, KIRC: Kidney renal clear cell carcinoma, KIRP: Kidney renal papillary cell carcinoma, LIHC: Liver hepatocellular carcinoma, LUSC: Lung squamous cell carcinoma, OV: Ovarian serous cystadenocarcinoma, READ: Rectum adenocarcinoma, SKCM: Skin Cutaneous Melanoma, STAD: Stomach adenocarcinoma, THYM: Thymoma, UCEC: Uterine Corpus Endometrial Carcinoma.

RASD1 mRNA and protein are present in most tissue types in normal human tissues (Figure 1a,b and Supplementary Figure S1). Across normal tissues, RASD1 mRNA levels are typically higher in the pituitary gland, mammary tissue, and adipose tissue and lower in cultured fibroblasts, lymphocytes, and whole blood (Figure 1a). Conversely, RASD1 protein expression is notably higher in digestive organs while absent in smooth muscle and adipose tissue (Figure 1b). Similarly, RASD1 mRNA and protein are present in a variety of cancer types. (Figure 1c,d). Notably, colorectal cancers exhibited high protein expression (Figure 1e), while lymphomas (Figure 1f), gliomas (Figure 1g), and pancreatic cancer (Figure 1h) displayed low to no protein expression of RASD1. Furthermore, RASD1 mRNA is generally downregulated in most cancers compared to normal tissue; however, it exhibits upregulation in thymoma (THYM) and uterine corpus endometrial carcinoma (UCEC) (Figure 2).

Since cell lines are commonly used in cancer biology research to study molecular mechanisms and testing of therapeutic agents, we examined RASD1 expression in cancer cell lines. Similarly to the expression in tumor samples, RASD1 is expressed in most colorectal cancer cell lines and is either not present or is expressed in low levels in most brain, pancreatic cancer, and lymphoma cell lines (Supplementary Figure S2). However, there is significant variability within brain cancer cell lines (Supplementary Figure S2b).

### 2.2. RASD1 Promoter Methylation Is Low in Many Cancers

**Figure 3:**
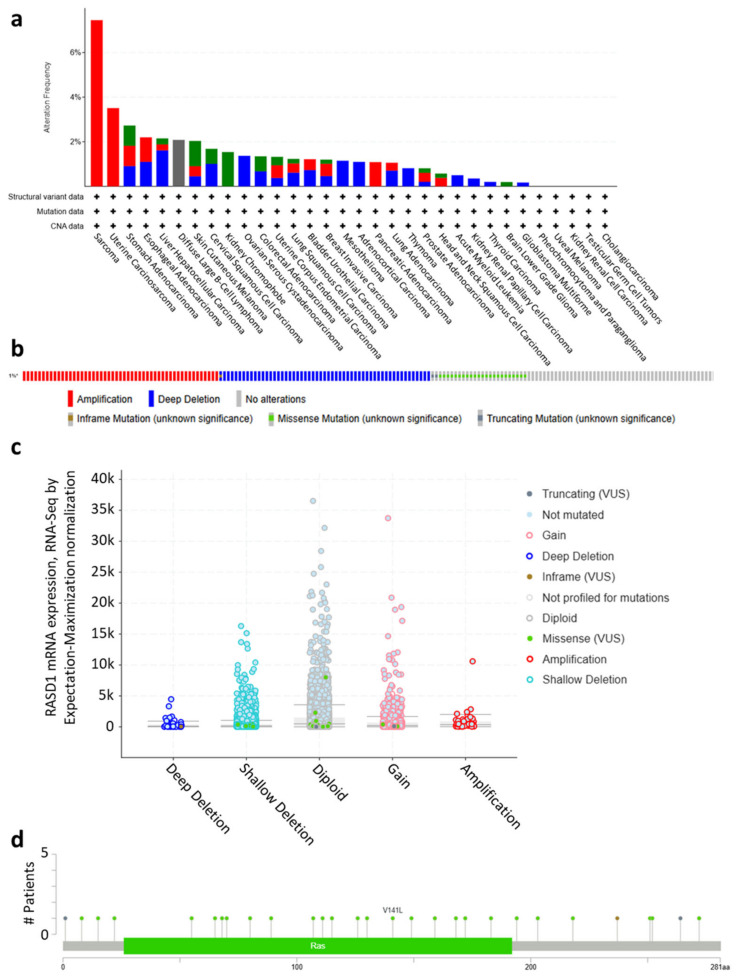
The genetic alterations of RASD1 (**a**) Alteration summary of RASD1 in TCGA pan-cancer datasets. Red: amplification; blue: deep deletion; green: mutation; gray: multiple alterations. (**b**) Summary of RASD1 structural variant, mutations, and copy-number alterations. * = not all samples are profiled. (**c**) The copy number alteration types of RASD1 in pan-cancer. (**d**) The point mutation sites of RASD1.

Genetic alterations may disrupt normal gene functions, potentially leading to cancer development if the gene is involved in cell growth and survival [18,19]. Therefore, we investigated the copy numbers and mutations of RASD1 across all cancers in the TCGA database to assess potential factors that could be influencing the regulation of RASD1 expression. Patients with sarcoma, esophagogastric cancer, hepatobiliary cancer, and mature B-cell neoplasms exhibited the highest rate of gene alterations in RASD1 (>2%) when compared to other cancer types (Figure 3a). The predominant genetic alterations in RASD1 included amplification, deep deletion, and missense mutations (Figure 3b). However, the copy number of RASD1 remains diploid in most samples (Figure 3c), suggesting that the variation in copy number may not be the main factor for regulating RASD1 expression in cancer. Additionally, there were no recurrent missense mutations detected in the *RASD1* gene (Figure 3d). Given that missense mutations typically affect protein function by altering amino acid sequences, this absence suggests that RASD1 regulation is likely to occur through other mechanisms.

**Figure 4:**
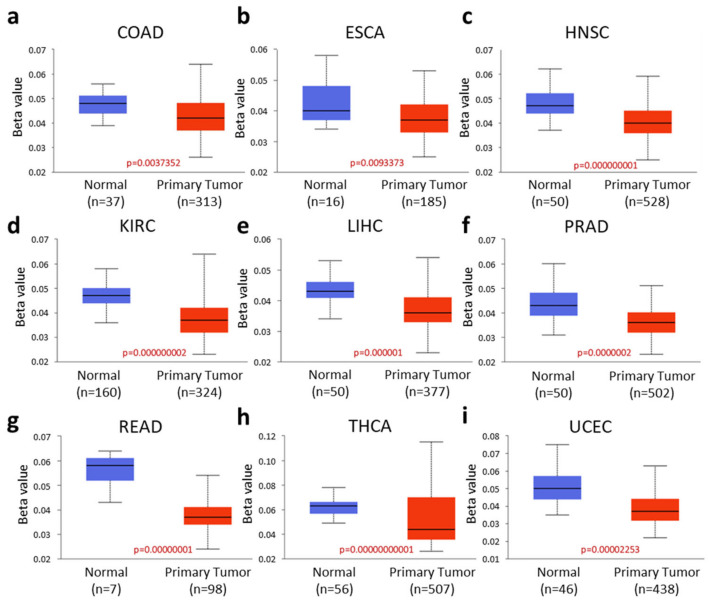
The promoter methylation levels of RASD1 are significantly lower in cancers. The promoter methylation levels of RASD1 in (**a**) COAD, (**b**) ESCA, (**c**) HNSC, (**d**) KIRC, (**e**) LIHC, (**f**) PRAD, (**g**) READ, (**h**) THCA, and (**i**) UCEC. COAD: Colon adenocarcinoma, ESCA: Esophageal carcinoma, HNSC: Head and Neck squamous cell carcinoma, KIRC: Kidney renal clear cell carcinoma, LIHC: Liver hepatocellular carcinoma, PRAD: Prostate adenocarcinoma, READ: Rectum adenocarcinoma, THCA: Thyroid carcinoma, UCEC: Uterine Corpus Endometrial Carcinoma. Data obtained from The University of ALabama at Birmingham CANcer data analysis Portal (UALCAN) (http://ualcan.path.uab.edu/, accessed on 12 September 2023).

Hypermethylation-driven transcriptional activation of oncogenes has been observed in tumorigenesis and metastasis [20]. We observed significantly lower promoter methylation levels of RASD1 in colon adenocarcinoma (COAD), esophageal carcinoma (ESCA), head and neck squamous cell carcinoma (HNSC), KIRC, LIHC, prostate adenocarcinoma (PRAD), READ, thyroid carcinoma (THCA), and UCEC compared to normal tissues (Figure 4a–i). These results suggest that RASD1 expression in many cancers may be regulated by reduced promoter methylation but not genetic alterations and its role as a tumor suppressor.

### 2.3. High RASD1 Expression in Tumors Correlates with Longer Survival in KIRC, LGG, and PAAD

**Figure 5:**
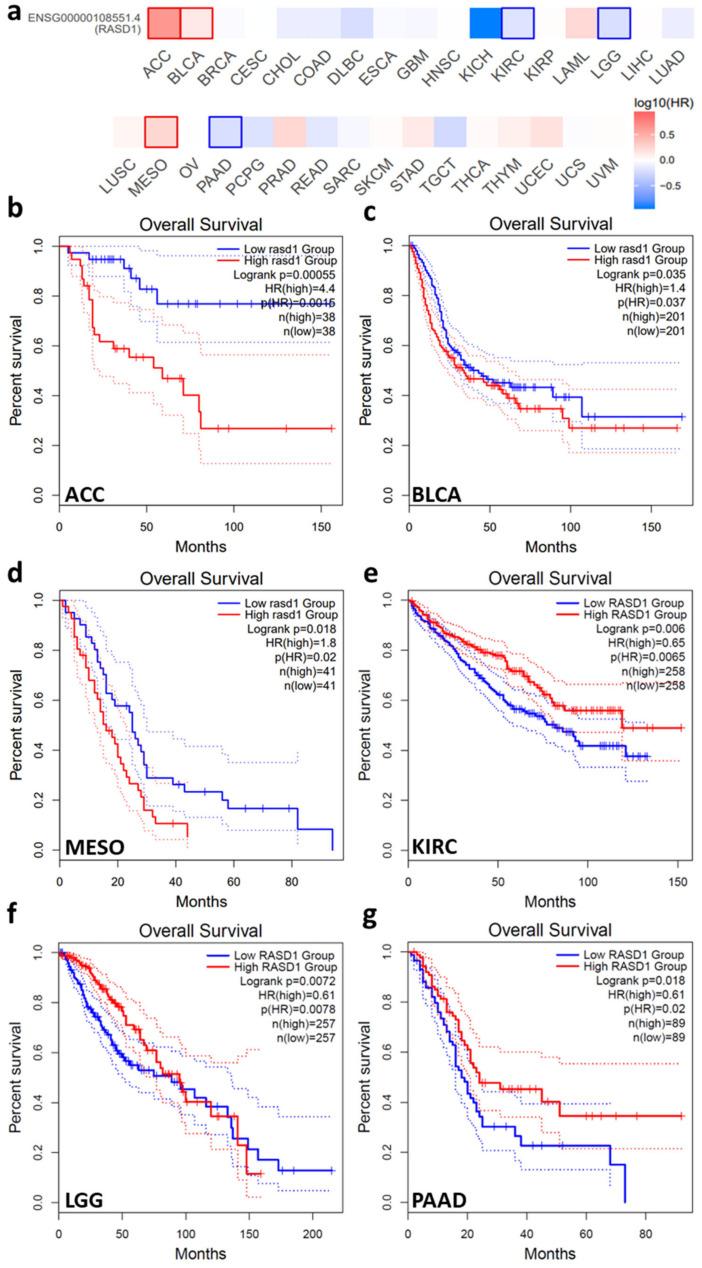
Correlation between RASD1 expression and overall survival in patients with different tumor types. GEPIA2 was used to perform overall survival analysis. (**a**) The overall survival significance map of RASD1 in pan-cancer. Kaplan–Meier analysis of the association between RASD1 expression in (**b**) ACC, (**c**) BLCA, (**d**) MESO, (**e**) KIRC, (**f**) LGG, and (**g**) PAAD.

The varied expression patterns of RASD1 across different cancer types suggest a potential role in patient outcomes. Therefore, we examined if there was a correlation between RASD1 expression levels and the overall survival of cancer patients (Figure 5a). The expression of RASD1 inversely correlated to survival outcomes in Adrenocortical carcinoma (ACC), Bladder Urothelial Carcinoma (BLCA), and Mesothelioma (MESO) (Figure 5b–d). In patients with KIRC, low grade glioma (LGG), and PAAD, high RASD1 expression is associated with prolonged survival (Figure 5e–g). Notably, RASD1 expression is lower in cancer tissue than in normal tissue in KIRC, and its protein expression is low in LGG and PAAD. These findings suggest a potential tumor-suppressing function of RASD1 in some cancers like KIRC, LGG, and PAAD.

### 2.4. RASD1 Expression Correlates with the Infiltration of CD4^+^ T Cells and Myeloid Dendritic Cells in KIRC, LGG, and PAAD

**Figure 6:**
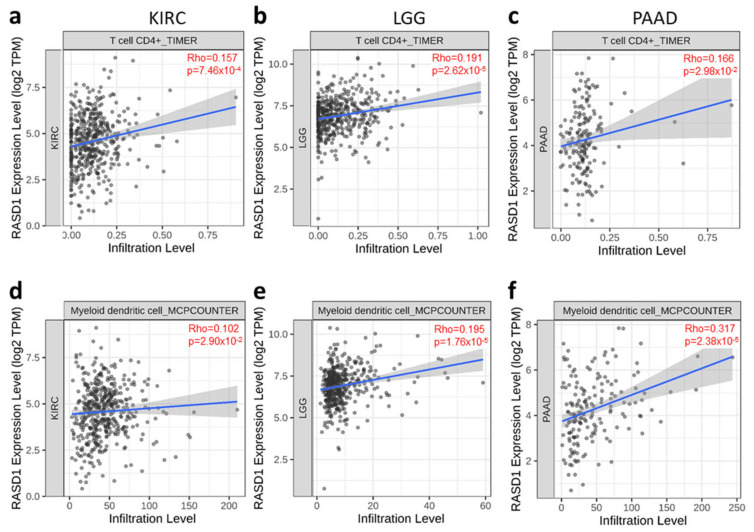
Correlation between RASD1 expression and immune cell infiltrates in KIRC, LGG and PAAD cancers. TIMER algorithm was used to calculate the correlation between RASD1 expression and CD4^+^ T cells in (**a**) KIRC, (**b**) LGG, and (**c**) PAAD. MCPCOUNTER algorithm was used to calculate the correlation between RASD1 expression and myeloid dendritic cells in (**d**) KIRC, (**e**) LGG, and (**f**) PAAD.

Previous studies have established a correlation between immune cell infiltration and the sensitivity to immunotherapy and survival [21,22]. Furthermore, RASD1 expression can be induced by dexamethasone, a common steroid used in cancer patients that can reduce inflammation. Therefore, in an exploratory manner, we aimed to further investigate this correlation and explore whether RASD1 expression in KIRC, LGG, and pancreatic adenocarcinoma (PAAD) is related to changes in the tumor microenvironment. We utilized the TIMER and MCPCOUNTER algorithms in TIMER2.0 to examine the association between immune cell infiltration and RASD1 expression (Figure 6). Our analysis revealed a positive correlation between RASD1 expression and CD4^+^ T cells (Figure 6a–c) and myeloid dendritic cells (Figure 6d–f) in KIRC, LGG, and PAAD. These findings suggest that RASD1 might play a role in modulating the immune response and tumor microenvironment of KIRC, LGG, and PAAD.

To further investigate the cancer-specific role of RASD1, we extended our analysis to ACC, BLCA, and MESO, in which RASD1 expression was inversely correlated with patient survival, using the same algorithms. In ACC, BLCA, and MESO, no significant association was observed between RASD1 expression and CD4⁺ T cell infiltration (Supplementary Figure S3a–c). Similarly, RASD1 expression did not correlate with myeloid dendritic cell infiltration in ACC and MESO (Supplementary Figure S3d,f). However, a positive correlation between RASD1 expression and dendritic cell infiltration was detected in BLCA (Supplementary Figure S3e). These findings highlight the cancer-type-specific nature of RASD1′s involvement in modulating the tumor immune microenvironment and suggest that its functional role may vary substantially across different cancers.

### 2.5. Enrichment Analysis of Genes with Similar Expression Patterns as RASD1 Suggests a Potential Involvement in Interleukin-4-Mediated Apoptosis and the Transcriptional Regulation of PTEN

**Figure 7:**
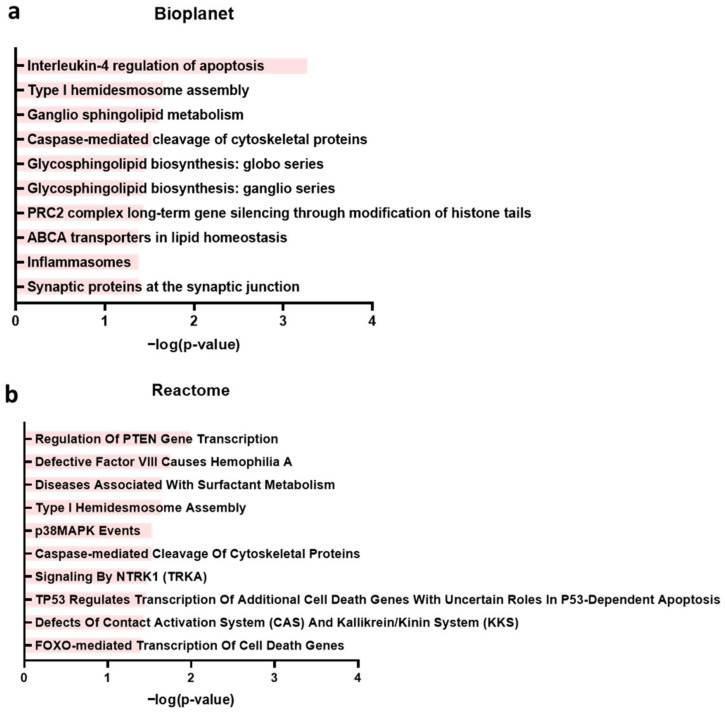
Enrichment analysis of RASD1-related genes in KIRC, LGG, and PAAD. (**a**) Bioplanet and (**b**) Reactome pathway analysis.

To explore the potential tumor suppressive mechanisms of RASD1 in KIRC, LGG, and PAAD, we used GEPIA2 to identify the top 50 genes (Table 1) with similar expression patterns as RASD1 in these three cancers for which high RASD1 expression correlated with better survival outcomes. The gene list was then passed through Enrichr to explore the functional associations and pathways linked to RASD1 expression in these cancer types (Figure 7). BioPlanet data showed that the genes with the highest correlation with RASD1 are mainly involved in “Interleukin-4 regulation of apoptosis” (Figure 7a). Furthermore, the Reactome pathway analysis indicates that RASD1 and genes associated with RASD1 expression may be involved in the “Regulation of PTEN gene transcription” during tumor pathogenesis (Figure 7b). Using genes with similar expression patterns to that of RASD1 sheds light on potential molecular mechanisms and pathways associated with RASD1 expression that may impact tumor biology.

## 3. Discussion

In this Pan-cancer analysis study, we observed distinct expression patterns of RASD1 across different tissues and cell types. Notably, RASD1 protein expression was detected in about 80% of colorectal cancer patients, whereas it was largely absent in patients with lymphoma and glioma according to data from the Human Protein Atlas. Moreover, RASD1 RNA expression was consistently lower in cancer tissues compared to normal tissues across several malignancies, except in THYN and UCEC, where it was upregulated. We found minimal genetic alterations in RASD1; however, the promoter methylation levels of RASD1 are lower in several cancer types, suggesting that low promoter methylation might predominantly regulate its expression in cancer. Importantly, high RASD1 RNA expression correlated with improved survival in KIRC, LGG, and PAAD and our finding suggests that this could potentially be due to increased infiltration of CD4^+^ T cells and myeloid dendritic cells in the tumor microenvironment. Enrichment analysis highlighted RASD1′s potential involvement in interleukin-4-mediated apoptosis and transcriptional regulation of PTEN. Therefore, RASD1 may modulate immune infiltration, in line with previous research on its potential role in promoting apoptosis [11,12]. Collectively, these findings offer valuable insights into RASD1′s potential role in tumor regulation.

While RASD1 expression generally remains low in most cancers compared to normal tissues, we observed the opposite in THYM, a cancer with a low incidence of KRAS mutations, and UCEC, a cancer in which patients with KRAS mutations exhibit a more favorable prognosis [23]. This suggests that the regulation of RASD1 may vary depending on tissue type, leading to the observed increase in expression in THYM and UCEC. This could potentially unveil novel associations of signaling pathways with RASD1 biology. For example, there was variable RASD1 expression across different cancer cell lines, with particularly notable variability between brain cancer cell lines. This range in RASD1 expression likely stems from inherent genetic and molecular distinctions characteristic of each specific cancer type, as well as the heterogeneity of tumors like pancreatic cancer, renal cancer, and glioblastoma [24,25,26]. Understanding these differences is crucial, as the presence or absence of RASD1 expression may differentially regulate cancer cell behavior and progression.

The discrepancies between RASD1 mRNA and protein levels across various tissues and cancer types suggest complex post-transcriptional and post-translational regulations [27]. For instance, in adipose tissue, high mRNA levels with undetectable protein indicate possible translational repression or rapid protein degradation [28]. Conversely, high RASD1 protein levels in digestive tissues despite only modest mRNA expression suggest enhanced translation efficiency or increased protein stability. Interestingly, its elevated protein expression in colorectal cancer implies a potential context-specific role that may support tumor development or maintenance. Previous in vitro studies have demonstrated that RASD1 possesses tumor-suppressive potential, such as limiting colony formation, migration, and invasion, in various cancers, including in lung, prostate, breast, gastric cancer and glioma [9,10,11,12,29]. In both lung and prostate tissues, RASD1 mRNA and protein are expressed at moderate to low levels, suggesting coordinated regulation. In breast cancer, RASD1 shows low transcript levels alongside high protein expression, indicating enhanced protein stability or translational regulation. In contrast, gliomas exhibit a notable disconnect between mRNA and protein levels, potentially reflecting post-transcriptional or post-translational mechanisms that limit RASD1′s functional activity, such as by KIAA1429, a regulatory subunit of the N6-methyladenosine methyltransferase, in gastric cancer [29]. Despite detectable protein expression in breast, lung, and prostate tissues, consistent tumor-suppressive effects are not observed, implying that additional context-specific factors may modulate RASD1′s role in tumor progression. It is also important to note that according to the Human Protein Atlas, immunohistochemistry (IHC) data for RASD1 remains pending external validation, due to low consistency between staining patterns and RNA expression, and the reliability score for the IHC data is currently uncertain [30]. This further emphasizes the need for integrated transcriptomic and proteomic analyses to accurately interpret the biological and pathological functions of RASD1 in future studies.

High levels of promoter methylation can inhibit the binding of transcription factors in cancer, thereby suppressing gene expression [31,32]. Although we observed lower levels of promoter methylation of RASD1 in cancer compared to normal tissues, the expression of RASD1 increases in ESCA, HNSC, PRAD, THCA, and UCEC. This could be explained by the fact that promoter methylation is not the only factor that regulates gene expression. Other factors include the binding of transcription factors [33], histone modifications [34], and the presence of antisense microRNAs [35]. Additionally, tissue-specific mechanisms can influence gene expression independently of methylation status [36]. Thus, while a general correlation exists between lower promoter methylation and gene expression, our findings suggest a complex regulation of RASD1 in cancer and only partially explains the range in RASD1 expression across cancer types.

Genetic alterations within genes can function as critical biomarkers for cancer diagnosis and prognosis [37]. In our analysis, we identified copy number variation as the most common genetic alteration of RASD1, particularly in sarcoma. However, this alteration is observed in only a small percentage of samples, with less than 2% in most tumor types and under 10% in sarcoma. Furthermore, RASD1 remains diploid in most cases, indicating that copy number variation may not be a significant determinant of RASD1 expression or its involvement in cancer development across various tumor types [38,39]. Other Ras-related oncogenes, like KRAS, have several prominent missense mutations [40,41]. Unlike other Ras-related oncogenes, we did not find any recurrent missense or nonsense mutations in RASD1, which implies that the detected mutations may not be directly associated with cancer [42]. Overall, the infrequent occurrence of copy number alterations and the absence of recurrent predicted loss-of-function mutations across various cancer types suggest that genetic alterations in RASD1 may not play a significant role in modulating RASD1 expression or activity in the cancers analyzed.

In contrast to the paradigm where promoter hypermethylation serves as a mechanism for transcriptional silencing, hypermethylation of the promoter region has been associated with the elevated expression of oncogenes [43]. In our study, we observed relatively low promoter methylation of RASD1 in cancer tissues compared to normal tissues, suggesting that RASD1 may function as a tumor suppressor gene. Additionally, the changes in beta values indicate generally low levels of methylation across the samples studied. This suggests that methylation patterns may not play as significant a role in gene regulation in these tumors as previously thought, or that other epigenetic modifications could be more influential in regulating gene expression. Interestingly, we observed low methylation levels in UCEC tumors and high expression in cancer tissues, which typically aligns with the idea of promoter DNA methylation driving transcriptional silencing. This discrepancy implies that different regulatory mechanisms may be operating in UCEC tumors.

While our analysis showed that RASD1 mRNA downregulation is a common feature in multiple cancers including KIRC, ACC, and BLCA, its prognostic implications are not the same. In KIRC, low RASD1 expression is significantly associated with poor patient prognosis, whereas the opposite association is observed in ACC and BLCA, where lower RASD1 expression correlates with better prognosis. These findings indicate that the biological consequences of RASD1 downregulation are cancer-type specific, and its role in tumorigenesis may be influenced by tumor-specific factors, such as genetic alterations or microenvironmental conditions. Thus, while RASD1 downregulation may be a common feature of these cancers, its functional relevance appears to be context-dependent, emphasizing the need for further studies to elucidate the underlying mechanisms governing its differential impact on cancer prognosis.

To explore possible tumor extrinsic and intrinsic factors by which RASD1 levels could potentially influence patient survival outcomes, we sought to compare immune cell infiltrates and gene set enrichments that were different between RASD1 high vs. low tumors. Using enrichment analysis, we identified several pathways that might be related to the tumor suppressor function of RASD1, such as the interleukin (IL)-4′s regulation of apoptosis. IL-4 is an important cytokine that mediates the differentiation of naive CD4^+^ T cells into CD4^+^ Th2 effector cells [44], maturation and survival of B cells [45], and immunoglobulin isotype switching [46]. Furthermore, IL-4 triggers apoptosis in acute myeloid leukemia cells through a Stat6-dependent pathway. This finding suggests that when RASD1 is present, it might help inhibit KRIC, LGG and PAAD by inducing cell death and modulating IL-4-related pathways. Another pathway that we found to have a strong association with RASD1 expression was the “Regulation of PTEN gene transcription”. PTEN is a tumor suppressor gene that regulates cell growth and survival by inhibiting the PI3K/AKT pathway [47]. PTEN is frequently inactivated in cancers and leads to abnormal cell growth in various cancers including prostate cancer, breast cancer, and colorectal cancer [48,49,50]. Moreover, PTEN mutations are commonly observed in various cancers [51], while germline PTEN mutations cause syndromes that lead to increased cancer risk [52]. Although RASD1 has been associated with inhibiting the AKT signaling pathway [10], a direct connection between RASD1 and PTEN has not been studied. It is possible that these two genes collaboratively inhibit the PI3K/AKT signaling pathway and inhibit cell growth and survival. Our enrichment analyses highlight potential mechanisms of how high RASD1 expression may negatively regulate cancers such as KIRC, LGG, and PAAD.

Previous studies have highlighted the various roles of RASD1 in multiple cancer types, functioning as both a tumor suppressor and an oncogenic regulator. In glioma, RASD1 has been demonstrated to act as a tumor suppressor, with overexpression inhibiting tumor invasion and progression [10]. This aligns with our findings that patients with low RASD1 expression exhibit poorer prognoses. In breast cancer, RASD1 has been implicated in apoptosis regulation via the mitochondrial pathway, where its upregulation induces apoptotic cell death [11]. Notably, our analysis revealed reduced RASD1 mRNA levels in breast cancer tissues, suggesting a potential loss of its apoptotic regulatory function in tumorigenesis. Similarly, in gastric cancer, KIAA1429, a regulatory subunit of the m6A methyltransferase complex, promotes tumor growth and metastasis by destabilizing RASD1 mRNA through an m6A-YTHDF2-dependent mechanism [29]. Our findings support this observation by showing low RASD1 mRNA levels in stomach cancer tissues. Conversely, prior studies have reported that elevated RASD1 expression correlates with poor survival and chemotherapy resistance in B-cell acute lymphoblastic leukemia (B-ALL) [53]. Unfortunately, due to the lack of B-ALL data in the TCGA dataset, we were unable to perform an analysis between RASD1 and B-ALL. These findings highlight the complexity of RASD1′s role in cancer biology, emphasizing its tissue-specific functions and clinical relevance.

In recent years, significant progress in RAS-targeted therapies has opened new avenues for cancer treatment [54]. Among RAS isoforms, KRAS is particularly well known for its central role in reprogramming cancer metabolism to support tumor growth and survival [55]. In our enrichment analysis, we show that RASD1-related genes are involved in both apoptotic processes and metabolic pathways. These findings suggest that RASD1 may impact tumor biology not only by promoting cell death but also through modulation of metabolic processes. With growing interest in targeting metabolic pathways in RAS-driven cancers, RASD1 presents a promising candidate for therapeutic intervention, either independently or in combination with existing metabolic or apoptotic strategies.

There are several limitations of this pan-cancer analysis. First, the small sample sizes for some of the less prevalent cancers pose a challenge for drawing robust conclusions, as they limit the statistical power of the analysis, impairing the ability to distinguish significant associations or distinctions between tumor types. This limitation affects the reliability of observed associations and makes it challenging to generalize findings to broader patient populations. Second, the analysis relies heavily on publicly available datasets that may introduce biases related to sample selection, preprocessing methods, and population representation. These factors could potentially influence the observed patterns of RASD1 expression and its associations with clinical outcomes. Third, while this study highlights correlations between RASD1 expression and patient survival, the findings are preliminary and require validation in larger, independent cohorts. We acknowledge that this study cannot prove the molecular functions of RASD1 in tumor suppression. Subsequent experimental work is needed to unravel the precise role of RASD1 in the complex landscape of cancer development and patient survival across cancers. However, this study highlights the need for further exploration to elucidate the specific molecular mechanisms RASD1 influences in different cancer contexts. Finally, differences in bioinformatics algorithms and parameter settings across various platforms could lead to inconsistencies in data interpretation, underscoring the need for careful methodological standardization. Despite these limitations, the study offers valuable insights into RASD1′s potential role in cancer and highlights the need for focused experimental investigations to unravel its precise functions and therapeutic implications.

In conclusion, RASD1 is commonly expressed in various tissues and exhibits lower expression in most cancer types, often related to poor clinical outcomes. The analysis of immune infiltration and enrichment of genes associated with RASD1 suggests potential mechanisms by which RASD1 could influence tumor immunity, genetic alterations, and signaling pathways in cancers. These findings suggest exciting possibilities for future research focused on how RASD1 signaling may contribute to the biology of various tumors.

## 4. Materials and Methods

RASD1 expression analysis

RNA and protein expression of RASD1 in normal and cancer tissues were acquired using the GTEx portal (https://www.gtexportal.org/home/, accessed on 5 August 2023) [16] and the Human Protein Atlas database (https://www.proteinatlas.org/ accessed on 17 September 2023) [17]. The query “RASD1” was used to access RNA expression data from the GTEx portal and the Human Protein Atlas website. To compare RASD1 expression in normal and tumor tissues across 33 cancer types, RNA data were analyzed using the Gene Expression Profiling Interactive Analysis (GEPIA2) website (http://gepia2.cancer-pku.cn/, accessed on 24 August 2023) [56]. In GEPIA, box plots comparing the TCGA normal and GTEx data for 33 cancer types was performed using the “Expression DIY” function.

Genetic alteration of RASD1 in pan-cancer

The cBioPortal website (https://www.cbioportal.org/, accessed on 17 September 2023) [57] was used to analyze the alteration frequency, mutation types, and copy number aberrations (CNAs) of RASD1 across all cancer types in The Cancer Genome Atlas (TCGA) [14]. Data were obtained from the “OnCoprint”, “Cancer Types Summary”, “Plots”, “Mutations”, and “Genomic Alterations” within the “Comparison/Survival” module. Additionally, The University of ALabama at Birmingham CANcer data analysis Portal (UALCAN) (http://ualcan.path.uab.edu/ 17 September 2023) [58] was utilized to explore the promoter methylation levels of RASD1 across cancers. To access the data, the query “RASD1” was used in the TCGA tab of the website and “Methylation” was chosen to view the RASD1 promoter methylation profile categorized by sample types.

Survival analysis

The “Survival map” module in GEPIA2 [56] was used to obtain the overall survival (OS) significance map across all cancer types in the TCGA dataset. Additionally, Kaplan–Meier analysis was performed using the “Survival Analysis” module in GEPIA2 to evaluate the association between RASD1 expression and OS in each cancer type. Samples were divided into high and low-expression groups using the median as a cutoff.

Immune infiltration analysis

The immune association module in TIMER2.0 (http://timer.cistrome.org/, accessed on 17 September 2023) [59] was used to assess the association between RASD1 expression and the abundance of immune cell types in all cancers in TCGA. The TIMER and MCPCOUNTER algorithms were applied.

Gene enrichment analysis

“Similar Genes Detection” in GEPIA2 was used to identify the top 50 correlated genes exhibiting a similar expression pattern to RASD1 in KIRC, LGG, and PAAD, based on Pearson correlation coefficient (PCC). The selected gene list was then analyzed with Enrichr (http://amp.pharm.mssm.edu/Enrichr/, accessed on 1 January 2020) [60], with pathway enrichment analysis results obtained from The National Center for Advancing Translational Sciences (NCATS) BioPlanet and Reactome databases.

## Figures and Tables

**Table 1:** Top 50 RASD1-correlated genes in KIRC, LGG, and PAAD.

Gene Symbol	Gene ID	PCC ^1^
DLG5-AS1	ENSG00000233871.2	0.57
TMOD1	ENSG00000136842.13	0.56
SCARA3	ENSG00000168077.13	0.54
NLRP1	ENSG00000091592.15	0.53
PDE8B	ENSG00000113231.13	0.53
SMG6	ENSG00000070366.13	0.53
DST	ENSG00000151914.17	0.53
CADPS	ENSG00000163618.17	0.52
TMEM132E	ENSG00000181291.6	0.52
KCNH1-IT1	ENSG00000234233.1	0.52
HMBOX1	ENSG00000147421.17	0.51
FJX1	ENSG00000179431.6	0.51
FRMPD3	ENSG00000147234.10	0.51
RING1	ENSG00000204227.4	0.51
MED9	ENSG00000141026.5	0.51
TPST1	ENSG00000169902.13	0.5
SPEG	ENSG00000072195.14	0.5
ROM1	ENSG00000149489.8	0.49
C1QL1	ENSG00000131094.3	0.49
TCEAL3	ENSG00000196507.10	0.49
ATN1	ENSG00000111676.14	0.49
PRAF2	ENSG00000243279.3	0.49
NAV2	ENSG00000166833.19	0.49
C6orf1	ENSG00000186577.11	0.49
DCAKD	ENSG00000172992.11	0.49
ST8SIA1	ENSG00000111728.10	0.48
LRSAM1	ENSG00000148356.13	0.48
GALNT15	ENSG00000131386.17	0.48
SDR39U1	ENSG00000100445.16	0.48
JAKMIP2	ENSG00000176049.15	0.48
RP11-209D14.2	ENSG00000261033.1	0.48
ABCA3	ENSG00000167972.13	0.48
GAP43	ENSG00000172020.12	0.47
TCEA2	ENSG00000171703.16	0.47
TMEM200C	ENSG00000206432.4	0.47
SEC14L2	ENSG00000100003.17	0.47
ID4	ENSG00000172201.10	0.47
BAALC	ENSG00000164929.16	0.47
USP20	ENSG00000136878.12	0.47
PMP2	ENSG00000147588.6	0.47
ADD1	ENSG00000087274.16	0.47
BCL6	ENSG00000113916.17	0.47
FAM127A	ENSG00000134590.13	0.47
FAM167A	ENSG00000154319.14	0.47
BBS2	ENSG00000125124.11	0.47
FAM69B	ENSG00000165716.9	0.47
GFAP	ENSG00000131095.11	0.47
SPOCK2	ENSG00000107742.12	0.47
RALGDS	ENSG00000160271.14	0.46
BAALC-AS2	ENSG00000236939.2	0.46

^1^ PCC: Pearson correlation coefficient.

## Data Availability

These data were derived from the following resources available in the public domain: GTEx https://www.gtexportal.org/home/ accessed on 2 September 2023, Human Protein Atlas https://www.proteinatlas.org/ accessed on 2 September 2023, and TCGA https://portal.gdc.cancer.gov/ accessed on 2 September 2023.

## Supplementary Materials

The following supporting information can be downloaded at: https://www.mdpi.com/article/10.3390/ijms26094419/s1.

## Abbreviations

The following abbreviations are used in this manuscript:

RASD1	dexamethasone-induced Ras-related protein 1
TCGA	The Cancer Genome Atlas
GTEx	Genotype-Tissue Expression
PTEN	phosphatase and tensin homolog
MAPK	mitogen-activated protein kinase
PI3K	phosphoinositide 3-kinase
AKT	protein kinase B
mTOR	mammalian target of rapamycin
THYM	Thymoma
UCEC	Uterine corpus endometrial carcinoma
ACC	Adrenocortical carcinoma
BLCA	Bladder Urothelial Carcinoma
BRCA	Breast invasive carcinoma
CESC	Cervical squamous cell carcinoma and endocervical adenocarcinoma
COAD	Colon adenocarcinoma
KICH	Kidney Chromophobe
KIRC	Kidney renal clear cell carcinoma
KIRP	Kidney renal papillary cell carcinoma
LIHC	Liver hepatocellular carcinoma
LUSC	Lung squamous cell carcinoma
OV	Ovarian serous cystadenocarcinoma
READ	Rectum adenocarcinoma
SKCM	Skin Cutaneous Melanoma
STAD	Stomach adenocarcinoma
ESCA	Esophageal carcinoma
HNSC	Head and neck squamous cell carcinoma
PRAD	Prostate adenocarcinoma
THCA	Thyroid carcinoma
MESO	Mesothelioma
PAAD	Pancreatic adenocarcinoma
LGG	Low grade glioma
IL	Interleukin
B-ALL	B-cell acute lymphoblastic leukemia
