# Supplementary material for: Pan-Cancer Analysis Identifies a Ras-Related GTPase as a Potential Modulator of Cancer
Source: Int J Mol Sci. 2025 May 6;26(9):4419. doi: 10.3390/ijms26094419 (PMC12073092; doi:10.3390/ijms26094419)
Supplement: Supplementary file 1 [file ijms-26-04419-s001.zip › ijms-3543377-supplementary.pdf]

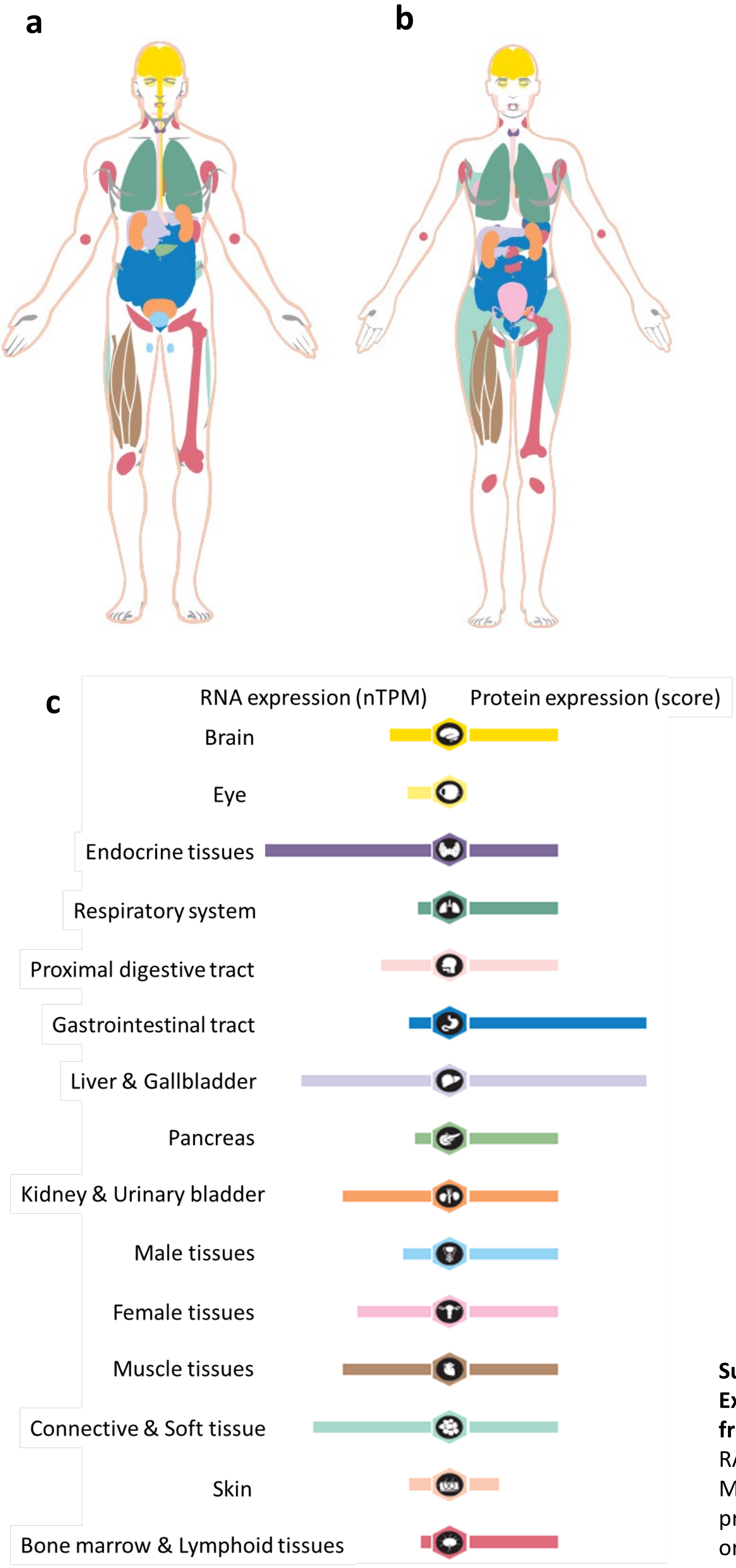

Supplementary Figure 1: Expression summary of RASD1 from Human Protein Atlas. RASD1 expression pattern in **(A)** Male and **(B)** Female. **(C)** RNA and protein expression summary by organs

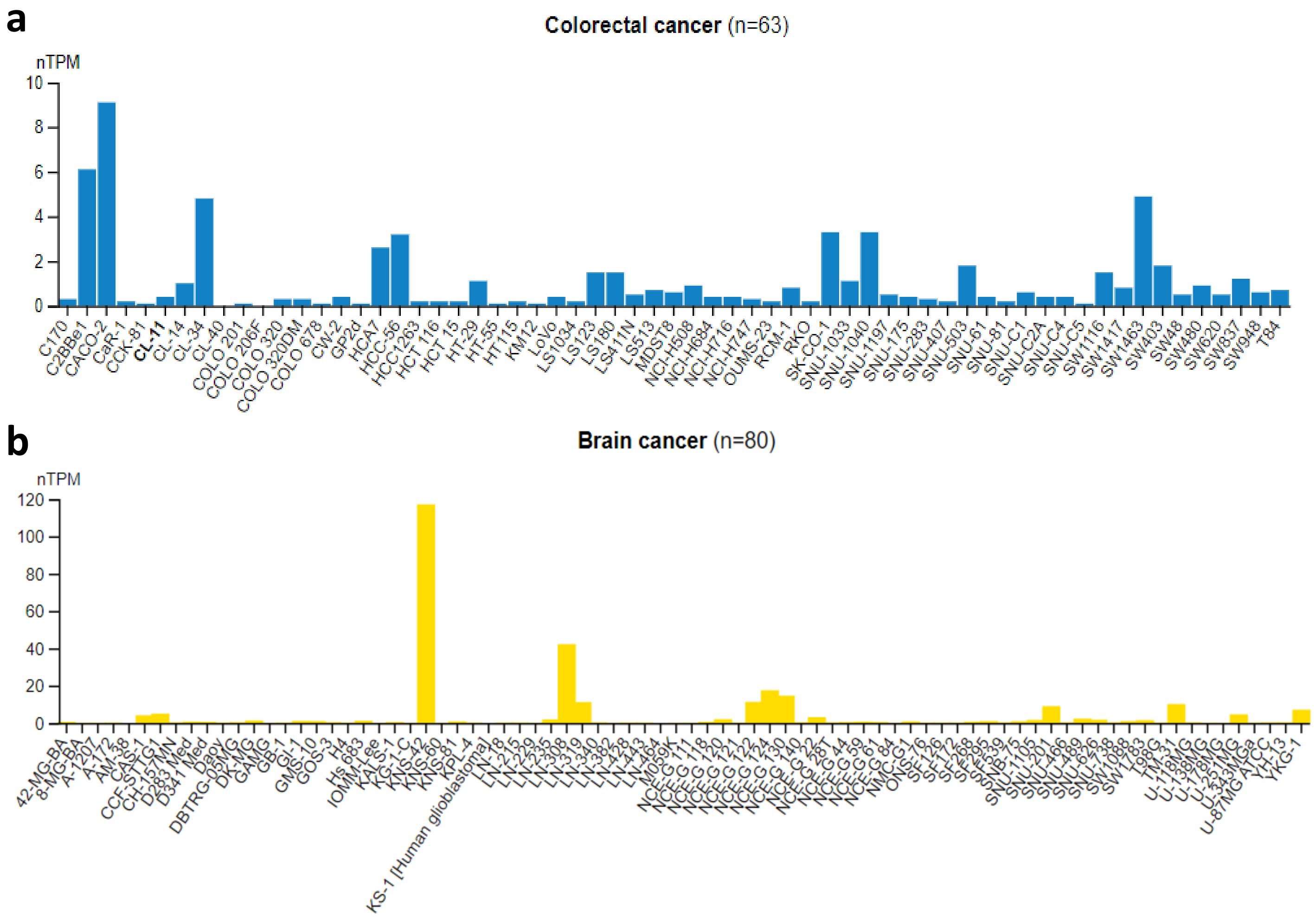

Supplementary Figure 2. RASD1 expression in cancer cell lines. The expression levels of RASD1 in cell lines obtained from the Human Protein Atlas. (A) colorectal cancer (B) brain cancer

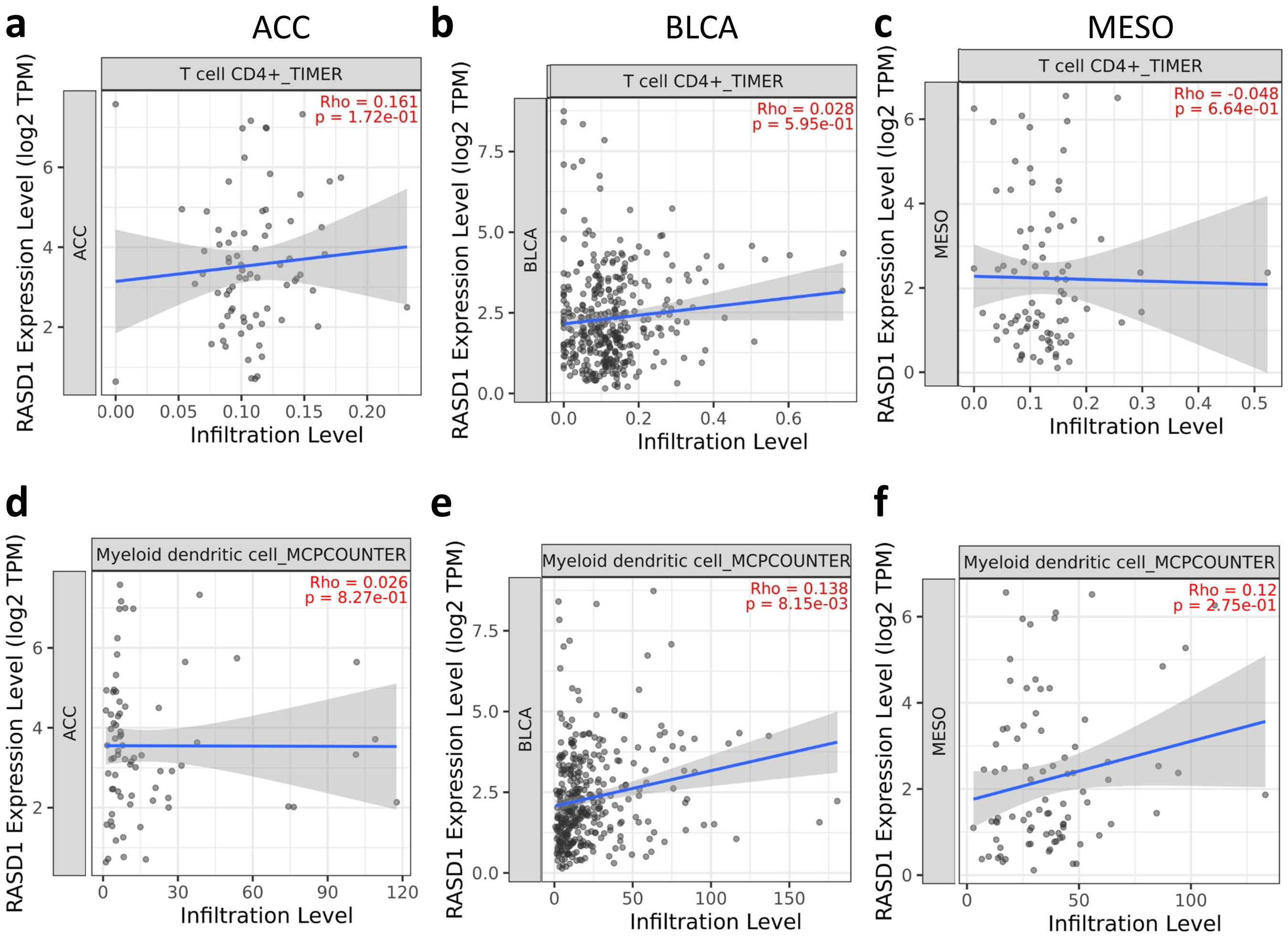

Supplementary Figure 3. Correlation between RASD1 expression and immune infiltrates in ACC, BLCA and MESO cancers. TIMER algorithm was used to calculate the correlation between RASD1 expression and CD4 T cells in **(A)** ACC, **(B)** BLCA, and **(C)** MESO. MCPCOUNTER algorithm was used to calculate the correlation between RASD1 expression and myeloid dendritic cells in **(D)** ACC, **(E)** BLCA, and **(F)** MESO.
